# Venous Thromboembolism Risk Assessment Audit

**DOI:** 10.1192/bjo.2025.10643

**Published:** 2025-06-20

**Authors:** Sunday Neru, Chimdimma Ozongwu, Ikrom Arzi, Shariffa Scerif, Jack Suen

**Affiliations:** North London NHS Foundation Trust, London, United Kingdom

## Abstract

**Aims:** This audit is most relevant to acute inpatients at a general psychiatric hospital (St Ann’s Hospital and Chase Farm Hospital) in which there is high turn over of acutely unwell psychiatric patients being admitted. This set of patients are at significant risk of venous thromboembolism due to immobility and the nature of their illnesses.

People with psychiatric disorders may be at risk of developing venous thromboembolism, particularly when acutely unwell and admitted to an acute psychiatric ward. This may be due to the presence of risk factors such as reduced mobility due to psychiatric illness or sedation, dehydration due to poor oral intake or comorbid physical illnesses. The use of antipsychotic medications also increases thrombotic risk. Parity of esteem for mental health is a priority for health care and should include equity of provision for the management of physical health problems in those people presenting primarily with mental illness.

Also, there are issues which may cause concerns with regard to VTE prophylaxis in this population such as capacity to consent to interventions, interactions of psychotropic medications with pharmacological thromboprophylaxis and risk issues around the use of pharmacological and mechanical strategies for people who self harm.

The aim of this audit is to find out if admitting doctors are complying with North London NHS Foundation Trust policy and Department of Health guidelines. This will be carried out looking at 40 admissions across 4 wards in St Ann’s Hospital and Chase Farm Hospital between 1/1/2023 to 30/6/2023.

**Methods:** Data collection using designed questionnaire.

Standards: Trust policy and Department of Health guideline to be used as standard.

**Results:** Out of the 40 admission cases viewed, none of them had VTE risk assessment done. Hence no data available to analyse.

**Conclusion:** It is unfortunate that doctors are no longer doing VTE risk assessment on an acute psychiatric ward. This is still being emphasized by Department of Health.

It is should be noted that about 25% of those who have pulmonary embolism die from it and DVT can lead to pulmonary embolism. It also should be noted that after the age of 40 years risk of VTE almost doubles every decade.

Recommendations:

Assess all acute psychiatric patients to identify risk of VTE and bleeding as soon as possible after admission to hospital or by the time of the first consultation review, using a tool published by a national UK body, professional network or peer-reviewed journal. The most commonly used risk assessment tool for hospital is the Department of Health risk assessment tool.

Reassess all people admitted to an acute psychiatric ward for risk of VTE and bleeding at the point of consultant review or if their clinical condition changes.

Consider pharmacological VTE prophylaxis with fondaparinux sodium if Low Molecular Weight Heparin is contraindicated for people admitted to an acute psychiatric ward whose risk of VTE outweighs their risk of bleeding.

Continue pharmacological VTE prophylaxis for people admitted to an acute psychiatric ward until the person is no longer at increased risk of VTE.

Action Plans:

This audit should be repeated in 12 months time.

This audit report should be discussed during induction programme in August this year.

This audit report should be shared with all consultants in the trust.

Nursing staff to check if admitting doctor has completed the VTE risk assessment as part of admission clerking.

Ward consultants including Dr Neru, ward managers and Dr C. Ozongwu (if available) to monitor the implementation of these recommendations.

